# Targeting the NuRD Component, CHD4, Impairs Foxp3+ Treg Cell Production and Function and Promotes Anti-Tumor Immunity

**DOI:** 10.64898/2026.07.30.741771

**Published:** 2026-07-31

**Authors:** Yan Xiong, Liqing Wang, Martina Minisini, Fanhua Kong, Eros Di Giorgio, Tatiana Akimova, Shane Horman, Ivan Babic, Elmar Nurmemmedov, Wayne W Hancock

## Abstract

Little is known about why Foxp3⁺ regulatory T (Treg) cells require at least three HDAC1/HDAC2-containing chromatin-remodeling complexes (NuRD, Sin3 and CoREST), or whether selective disruption of these complexes can be exploited to enhance antitumor immunity. Here, we investigated the role of chromodomain helicase DNA-binding protein 4 (CHD4), the ATP-dependent remodeling subunit of the NuRD complex, in Treg biology. Conditional deletion of Chd4 in Foxp3⁺ Tregs resulted in severe systemic autoimmunity and early lethality, accompanied by reduced Foxp3 expression, impaired Treg suppressive function, and loss of Treg lineage stability. Transcriptomic analyses demonstrated that CHD4 deficiency closely phenocopied Hdac2 deletion, whereas quantitative proteomic analyses revealed that CHD4 assembles into highly conserved NuRD complexes in both Treg and conventional CD4⁺ T cells. These findings indicate that the selective dependence of Tregs on CHD4 does not arise from the formation of lineage-specific protein complexes but rather from the unique epigenetic program maintained by CHD4-containing chromatin-remodeling complexes that is required for Treg differentiation and stability. Using a novel cellular target-engagement platform, we identified CH41, a potent small-molecule inhibitor of CHD4 that recapitulated the effects of genetic CHD4 ablation on Treg function. Pharmacological inhibition of CHD4 impaired intratumoral Treg accumulation and function and significantly inhibited the growth of lung and hepatocellular carcinomas in immunocompetent, but not immunodeficient, mice, without inducing systemic autoimmunity. Collectively, our findings identify CHD4 as a critical epigenetic regulator of Treg lineage stability and establish pharmacological targeting of the CHD4/NuRD axis as a promising strategy to selectively disrupt tumor-associated Tregs and enhance antitumor immunity.

## Full Text Availability

The license terms selected by the author(s) for this preprint version do not permit archiving in PMC. The full text is available from the preprint server.

